# Synthetic House-Tree-Person Drawing Test: A New Method for Screening Anxiety in Cancer Patients

**DOI:** 10.1155/2019/5062394

**Published:** 2019-03-26

**Authors:** Lijuan Sheng, Guifang Yang, Qian Pan, Chunfang Xia, Liping Zhao

**Affiliations:** ^1^Department of Neurology, The Second Xiangya Hospital, Central South University, China; ^2^Department of Emergency, The Second Xiangya Hospital, Central South University, China; ^3^Department of Oncology, The Second Xiangya Hospital, Central South University, China; ^4^Department of Nursing, The Second Xiangya Hospital, Central South University, China

## Abstract

The synthetic house-tree-person (S-HTP) drawing test is a projective measure primarily designed to assess specific complex personality traits. It is widely used in general psychological problems and mental illness such as psychological crisis intervention. Applicability and validity of S-HTP drawing test in cancer patients suffering from anxiety are still unclear and there are no reports on such research. The aim of this study was to explore the prevalence of anxiety in cancer patients and to investigate the applicability of S-HTP drawing test in such patients. Self-rating anxiety scale (SAS) and the S-HTP drawing test were applied to 167 cancer patients (58.7% male; 41.3% female), 52.92±10.43 years old. On SAS, anxiety rate was found in 16.17% cancer patients. Using the evaluation results from SAS as the dependent variable and the anxiety drawing characteristics as the independent variables, the logistic regression equation was established, and 9 drawing features were employed in the regression equation (*χ*2=56.982, P≤0.001, Nagelkerke R2=0.492). It is concluded that there is a positive correlation between S-HTP drawing test and SAS for anxiety state of cancer patients (p<0.01). S-HTP drawing test and SAS have interrater reliability and test-retest reliability. Our findings indicate that the S-HTP drawing test could help in screening anxiety in cancer patients.

## 1. Introduction

The incidence of cancer has been on the rise worldwide due to the increase in aging population and unhealthy lifestyle practices. Epidemiological studies have shown that cancer has become the leading cause of deaths in humans [1]. In China, the high incidence and mortality of cancer has become a major public health problem [2]. Most cancer cases are usually accompanied by severe pain, are often incurable, and require costly treatment. As a result, patients suffer from mental illnesses such as anxiety and depression and even have suicidal tendencies [3]. Anxiety is a feeling of uneasiness and worry, usually generalized and unfocused as an overreaction to a situation that is only subjectively seen as menacing. It is the subjectively unpleasant feelings of dread over anticipated events, such as the feeling of imminent death [4]. Recently, new medical models that incorporate psychological factors associated with the occurrence and development of cancer have drawn considerable attention. Numerous studies have confirmed that cancer patients have a high level of anxiety based on various measurement tools [5, 6]. About 18% to 20% of cancer survivors have anxiety issues, which not only reduce their quality of life of such patients but also significantly increase the medical costs and mortality [6]. In China, surveys on anxiety in cancer patients are mainly performed using questionnaires. However, in case where the patients have difficulties in expressing their inner feelings and thoughts and are not willing to cooperate, then using questionnaires alone produces unreliable results. Thus, projection testing can be an important supplement [7, 8] as it is one of the three major testing techniques used in psychology (questionnaire test, projection test, and situational test). This type of technique is able to transcend the subjects' psychological defenses to obtain inner thoughts and opinions. Synthetic house-tree-person (S-HTP) drawing test is a typical projection technique widely used in psychology. Compared to standardized tools commonly used to detect anxiety, the S-HTP drawing test can identify and reveal the inner emotions of cancer patients [9]. The set of items representing the drawing characteristics relating to the mood status of patients have already been collated, developed, and assessed by numerous scholars based on the drawing test manual and the related literature.

The synthetic-house-tree-person (S-HTP) test, which is a version of the HTP test, was developed by Buck in 1948 [10]. Mikami developed the S-HTP test in Japan in 1979 [11]. The original house-tree-person test (HTP) requires participants to draw three subjects on three pages. The special characteristic of the S-HTP test is that the house, the tree, and the person are drawn on the same sheet of paper and assessed together in relation to each other. Furthermore, the S-HTP is less of a burden because all of the objects can be drawn in the one sitting and can be taken in a relatively short time. Currently, the S-HTP drawing test is widely used for general psychological problems, mental illness such as schizophrenia, and psychological crisis intervention. The S-HTP drawing test not only reveals the discomfort of the patient in a bad situation but also improves self-awareness. Yet, it is rarely used in cancer patients. This study was designed to investigate its applicability and develop a predictive value that identifies anxiety in cancer patients.

## 2. Patients and Methods

*Subjects.* This cross-sectional study was performed in 200 participants from the Second Xiangya Hospital of Central South University, Changsha, Hunan, China, who agreed to join the study. 33 patients were excluded from the study due to failure to complete the S-HTP drawing test or the self-rating anxiety scale (SAS). Thus, the final sample consisted of 167 cancer patients.

*Inclusion and Exclusion Criteria.* The inclusion criteria were as follows: (1) having a pathological basis for cancer, (2) age ≥ 16 years, (3) ability to read and comprehend, and (4) agree to participate in the study. The exclusion criteria involved cancer patients with severe heart disease, liver disease, kidney dysfunction, respiratory failure, and poor physical fitness who were unable to complete the test. Patients who were familiar with the rules of the drawing test and those who had a professional foundation in drawing were excluded.

*Procedures.* All patients underwent a detailed in-person clinical interview. During this interview, the self-edited general information questionnaire, self-anxiety rating scale (SAS), and the S-HTP drawing test were performed. The patients first completed the S-HTP drawing test and then allowed to rest for 5-10 minutes. Afterwards, the self-edited general information questionnaire and self-anxiety rating scale (SAS) were performed.

*Self-Edited General Information Questionnaire.* The questionnaire consisted of 7 items including age, sex, nation, and so on (Table 1).

*Self-Rating Anxiety Scale (SAS) [12].* The SAS is a self-rating scale with 20 items. It includes five reverse-scoring items and 15 positive-scoring items that are rated on a 4-point scale from 1~4. The total score was obtained by adding all items, and the total score multiplied by a factor of 1.25 is the standard score of SAS. Based on Zung's classification criteria of levels of severity, psychiatry professionals in China have adapted SAS scores greater than 50 as the cutoff points for symptom severity associated with anxiety disorders, respectively, when conducting assessment, screening, or large-scale investigations in terms of Chinese National Normative Scores. The higher the score, the more severe the anxiety status, which is consistent with Zung's method. Both Zung's self-rating scales and the cutoff points described have been used extensively in studies of anxiety in China, and the Chinese translations of these surveys have been validated previously [13, 14].

*The S-HTP Drawing Test Applied [10, 15].* The whole process of S-HTP drawing test was done as follows: (1) tools: a piece of A4 white paper (210 mm × 297 mm), a 2B pencil, an eraser, and a writing board; (2) instructions: please draw a house, a tree, and a person and anything you want to draw in one picture. There is no time limit; (3) notes: the test must be completed by the individual. The patient should be informed that their drawing ability is not important and that they are just required to finish the drawing seriously. They cannot copy or use other tools. Any evaluation and tips cannot be given by researchers.

*S-HTP Drawing Test Scoring System [15–17].* We identified a set of drawing characteristics related to anxiety according to the R.C. Burns's drawing test interpretation system and other extant related literature. A total of 26 drawing features of anxiety were identified. These items were subsequently divided into the four domains of drawing characteristics (Table 2): (1) general S-HTP features included A1~A12, (2) house features included A13~A16, (3) tree features included A17~A22, and (4) person features included A23~A26. Each item was allocated a score of 1 or 0 if certain characteristics were found to be present or absent.

*Reliability and Validity Evaluation.* In this study, the reliability of the S-HTP test drawing features was evaluated using interrater reliability (Kappa coefficient) and test-retest reliability. The value of the Kappa coefficient ranged from 0.752 to 1.000. The value of the test-retest reliability r of 20 patients retested was [0.710, 0.857]. This indicated that the results of the drawing feature were stable and reliable. This study used the criterion validity to represent the S-HTP drawing test validity. Correlation analysis was performed using the data of anxiety from cancer patients identified by S-HTP drawing test and SAS scale (the result of SAS scale was regarded as the “gold standard”), and the correlation coefficient was the validity coefficient.

*Statistical Analysis.* All data in this study were analyzed using the SPSS program (SPSS 19.0, Chicago, IL, USA). Descriptive statistics (frequency, percentage, mean, and standard deviation) were used to classify and categorize data. An independent sample T-test was performed to compare the mean of SAS scores with that of the norm group. Chi-square test was used to examine different drawing characteristics in the anxiety group and nonanxiety group. The logistic regression equation was used to examine the role of S-HTP drawing characteristics in predicting anxiety. Statistical significance was set at P<0.05.

## 3. Results

*General Characteristics of Cancer Patients.* A total of 200 cases were enrolled in this study, among which 33 cases were excluded due to missing questionnaires or drawings. Finally, 167 cases were included, representing an effective rate of 83.5%. Among the 167 patients with cancer, 98 patients (58.7%) were male and 69 (41.3%) were female. Their age ranged from 16 to 72 years, with an average of 52.92±10.43 years. The patient's general characteristics are shown in Table 1.

*The Incidence of Anxiety among Cancer Patients.* In this study, the SAS standard score ranged from 25.00 to 63.75, with an average of 42.53 ± 7.72 points. The difference was statistically significant (t=14.613, P≤0.001) compared to the norm standard score [18] (the SAS norm standard is the result of X Liu's evaluation of 1097 normal Chinese people: where the standard score is 33.80±5.90). Twenty-seven people achieved a diagnostic score of anxiety of over 50 points. The incidence rate of anxiety among these cancer patients was 16.2%.

*Analysis of the Incidence of Various Items Used in Anxiety Assessment in Drawing Characteristics.* According to the drawing characteristics of the cancer patients, the incidence of the relevant evaluation items is shown in Table 2. Among the items, those with a higher incidence of anxiety status were A5, A1, A15, A3, and A9, suggesting that anxiety, restlessness, poor emotional control, sorrow, and annoyance are prevalent in cancer patients.

### 3.1. The Relationship between S-HTP Drawing Characteristics and Anxiety Status in Cancer Patients

*Univariate Analysis.* Considering the number of cases in this study and the number of S-HTP drawing characteristics (anxiety dimensions), we performed a univariate analysis according to the statistical theory. The *χ*2 test was performed on all 26 items in the anxiety group and nonanxiety group. The results revealed 12 different characteristics in the anxiety group and nonanxiety group. To capture all important factors, the features with P value ≤ 0.15 were included as independent variables in the logistic regression analysis. The details of these 12 drawing characteristics are shown in Table 3.

*Logistic Regression Analysis.* The results of the SAS were used as the dependent variable, setting the anxiety group to 1 and the anxiety-free group to 0. At the same time, 12 anxiety drawing characteristics (two categorical variables) which were selected by univariate analysis were used as independent variables to establish a logistic regression model (*α*_in_ = 0.10, *α*_out_ = 0.15). To investigate the relationship between the characteristics of S-HTP drawing test and anxiety in cancer patients, a total of 9 drawing characteristics were included in the equation. The total test results of the model coefficients showed that *χ*2=56.982, P≤0.001, indicating that the established logistic regression equation model was statistically significant. The regression equation for the characteristics of the S-HTP test on anxiety in cancer patients was established according to the regression coefficients shown in Table 4. Subsequently, the Nagelkerke R^2^ coefficient test was performed on the established regression equation. The regression equation established was as follows: Logit(P)=1.202-2.641*∗*A1-1.046*∗*A3+1.607*∗*A4+1.619*∗*A6-2.327*∗*A9+3.050*∗*A13+0.901*∗*A17+2.295*∗*A19-3.227*∗*A25. The Nagelkerke R^2^ value was determined to be 0.492, showing good fitness. This suggested that the equation had a good interpretation level, and the S-HTP drawing characteristics were suitable for assessing anxiety in cancer patients.

*The Results of S-HTP Drawing Test for Anxiety in Cancer Patients and Its Validity.* The logistic regression equation was employed to predict anxiety in 27 cancer patients who were diagnosed by the SAS scale. The findings demonstrated that 15 cancer patients had anxiety, with a correction rate of 55.6% (15/27). In 140 cancer patients without anxiety, the logistic regression equation predicted that 133 cancer patients did not have anxiety, and the correct rate was 95.0% (133/140). The overall correct rate was 88.6% (148/167), Table 5. In order to verify the validity of the S-HTP drawing test for anxiety in cancer patients, a correlation analysis was performed between the results of the S-HTP drawing test and SAS scale. The test results of the correlation coefficient of two detection methods $r=\left(ad-bc\right)/\sqrt{\left(a+b\right)\left(c+d\right)\left(a+c\right)\left(b+d\right)}=\left(15\times 133-12\times 7\right)/\sqrt{27\times 140\times 22\times 145}=0.55$, r>0.40, indicated good consistency between S-HTP test results and SAS results. Further consistency check was performed, *χ*2=[(|ad − bc| − n/2)2n]/[(a + b)(c + d)(a + c)(b + d)]=[(|15 × 133 − 12 × 7| − 167/2)2 × 167]/(27 × 140 × 22 × 145)=46.25. *χ*2>6.63, P<0.01 ($\chi^{\overset{2}{0.01,1}}$=6.63). We found that the results of the S-HTP drawing test for anxiety in cancer patients was positively correlated and consistent with those of the SAS scale.

*Univariate Analysis of SAS Scores in Different Subgroup.* Considering the anxiety levels vary with a wide range of other factors such as types of cancer, metastasis, pain degree, comorbidity disease, and stage of tumor, we performed a univariate analysis of SAS scores in different subgroup. It can be seen from the table that there is no difference in SAS scores in different subgroup analyses (*P*>0.05), Table 6.

## 4. Discussion

*Anxiety in Cancer Patients.* Anxiety refers to a feeling that adverse things may happen which are difficult to deal with resulting in some kind of nervousness, fear, and unpleasant emotions [19]. The data of this study reveal that the SAS standard score of the cancer patients was (42.53±7.72) points, which is significantly different compared to the normal group [18] (t=14.613, P≤0.001). This suggests that cancer patients are experience high level of anxiety that requires high attention. Twenty-seven cancer patients (16.2%) achieved a diagnostic score of the anxiety of over 50 points. The incidence of anxiety in cancer patients enrolled in this study is lower than that reported in previous studies in China. This may be attributed to the fact that not all subjects in this study had advanced cancer or the sample size was small.

*Applicability of S-HTP Drawing Test in Screening for Anxiety in Cancer Patients.* Most human thoughts and mental activities can be visualized. For example, the presence of physical objects can significantly enhance memory. Therefore, mental problems, such as anxiety, can be better identified and resolved by analyzing drawings. In China and other Asian cultures, individuals tend to be more conservative or withdrawn when sharing personal feelings or emotions [20, 21], and therefore, drawing can serve as an avenue for such individuals to express themselves.

*Analysis from the Aspect of the Incidence of Drawing Characteristic Items.* Five items related to anxiety in cancer patients with high incidence were as follows: (1) “A5 chaotic and curved lines” and “A15 slanted and curved wall lines”. In this study, 131 cancer patients (78.4%) had chaotic and curved lines (referring to the lines of the house, wall tree branches, or lines indicating messy hair). Among them, 113 (66.7%) had slanted and curved wall lines in their paintings. This indicated that cancer patients were anxious, emotionally uncontrollable, and depressed [22]. (2) “A1 very big drawing size”. A total of 113 cases (66.7%) had a very big drawing size (the area of the drawing was over 2/3 of the whole paper). The drawing size signifies the actual occupation of space or the desire to occupy space. Very big drawing size suggests that the patients are nervous and anxious about external stress [23]. (3) “A3 duplicated figures”. A total of 109 patients (65.3%) had duplicated figures in their paintings (referred to repeated figures on the line > 2 times). Handler L et al. pointed out that duplicated figures and shadows reflect anxiety [24]. (4) “A9 weak or heavy strength lines”. A total of 109 patients (65.3%) had weak or heavy strength in their drawing lines. Weak or heavy strength lines in drawings often reflect individual emotions and poor emotional control. When the above-mentioned drawing characteristics appear frequently, the cancer patients are considered to have anxiety and high stress levels.

*Analysis of the Result of the Established Logistic Regression Equation.* This study found that the correlation coefficient between the results of the S-HTP drawing test and the SAS scale in cancer patients was 0.55. The consistency hypothesis test showed that the S-HTP drawing test was associated with SAS (*χ*2=46.25, P<0.01). This indicated that the S-HTP test was applicable and effective in screening for anxiety in cancer patients. Furthermore, a similar finding was obtained from the established logistic regression equation. The nine drawing characteristic items in the logistic regression equation were as follows: (1) “A1, very big drawing size”. (2) “A3, duplicated figures”. (3) A4, colored black or shadow painting". Colored black or shadow painting in the drawing means that the whole or part of the drawing such as roofs, walls, and persons are colored black or shadow painted with a pencil. This is another form of anxiety expression in cancer patients [25]. (4) “A6, emphasizing the horizon”. Emphasizing the horizon at the bottom of the house, trees, among other objects with repeated lines means that the patient wants to escape or retreat. It is also a form of expression that emphasizes a sense of security implying that the painter may have anxiety [26, 27]. (5) “A9, weak or heavy strength lines”. (6) “A13, chimney”: the presence of a chimney in the drawing indicates that the painter wants to pursue warm interpersonal relationships and family support. This drawing feature is a positive trait. The absence of a chimney in the drawing suggests passiveness and lack of family support [28]. (7) “A17, discontinuous and curved lines in tree branches”. The discontinuous and curved lines in tree branches illustrate that the person is currently confused, which is directly due to challenges and underlying feelings [29]. (8) “A19, triangular tree branch”. In nature, a triangular tree branch is the result of natural evolution. It can resist wind and also fall, and the appearance of this drawing characteristic signifies that the person is mature and sophisticated. (9) “A25, no ears”. The absence of ears in the drawing demonstrate that the patient's self-intention is incomplete, which shows that the patient is an introvert and has low self-esteem. This often indicates that the patient has no desire to live [30]. It is noteworthy that many drawing evaluation items used in this study were not included in the regression equation. This is due to low frequency of such drawing characteristics or difficulty in comprehension. Although these S-HTP drawing test items may be related to anxiety, their predictability is less significant compared to the drawing characteristics items that correlate with the logistic regression equation.

Anxiety in cancer patients could be explain by diagnosis of the disease, poor therapeutic effect, and heavy economic burden of treatment. If anxiety exists and is not discovered early and managed effectively, it often results in severe consequences. Therefore, it is important to monitor the occurrence of anxiety in cancer patients and develop methods of evaluating it. The S-HTP drawing test can effectively reveal the psychological state of cancer patients as opposed to using the questionnaire alone since it can be conducted in less defensive situations [31]. Moreover, the S-HTP drawing test is a novel concept which allows the subject to exercise their creativity. In addition, it is an effective form of communication for researchers or patients who are not willing or unable to express their feelings verbally. If it is further standardized and improved with modern technology, the S-HTP drawing test will have more application prospects in the future [32].

## 5. Conclusion

According to the regression equation established from the drawing characteristics, the diagnostic results of anxiety in cancer patients were positively correlated with the results of the objective SAS scale. These findings revealed that the suitability of the two diagnostic methods for assessing anxiety was similar. Therefore, we can conclude that S-HTP drawing test is an effective tool for screening anxiety in cancer patients.

## 6. Limitations

There are some limitations in this study which must be taken into consideration. The narrow statistical population and small sample size are two shortcomings that may greatly influence the generalization of these results. The study is a cross-sectional descriptive study and would become an obstacle when finding causal explanations. Finally, these findings are based on Chinese patients; hence, it is unclear whether studies on other nationalities will yield similar findings.

## Figures and Tables

**Table 1:** Demographic characteristics of cancer patients.

Items	Characteristics	Cases	Constituent ratio (%)
Sex	Male	98	58.7
	Female	69	41.3
Nation	Han	161	96.4
	minority	6	3.6
Education	Elementary or junior	98	58.7
	Senior	44	26.3
	College or above	25	15.0
Marital status	Married	155	92.8
	Unmarried	12	7.2
Occupation	Civil servant or teacher	16	9.6
	Worker	22	13.2
	Farmer	71	42.5
	Businessman or freelancer	24	14.4
	Retired or unemployed	34	24.4
Income of the family per month (Ren Min Bi)	<1000	22	13.2
	1000~1999	76	45.5
	2000~2999	55	32.9
	3000~3999	10	6.0
	>4000	4	2.4

**Table 2:** The incidence of items used in anxiety assessment in drawing characteristics.

Item no.	Anxiety assessment items	Cases	Incidence (%)
A5	Chaotic and curved lines	131	78.4
A1	Very big drawing size	113	67.7
A15	Slanted and curved wall lines	113	67.7
A3	Duplicated figures	113	67.7
A9	Weak or heavy strength lines	109	65.3
A26	Angry expression on the face	104	62.3
A25	No ears	103	61.7
A21	Straight tree trunk	89	53.3
A2	Heavy strength figure	88	52.7
A10	Within the grids	73	43.7
A18	Swaying tree branches	69	41.3
A8	Thick or thin lines	66	39.5
A4	Colored black or shadow painting	60	35.9
A7	Excessive separation among house, tree, and person	58	34.7
A11	Repeatedly erased	53	31.7
A17	Discontinuous and curved line in tree branches	51	30.5
A12	Partitioning screen	50	29.9
A20	Fall down tree branches	48	28.7
A16	Roof drawn very carefully	41	24.6
A6	Emphasizes the horizon	38	22.8
A24	Chaotic lines of hair	38	22.8
A22	Leaves drawn very carefully	29	17.4
A23	Different size between persons	18	10.8
A19	Triangular tree branch	24	14.4
A13	Chimney	11	6.6
A14	Smoking chimney	5	3.0

**Table 3:** Drawing characteristics in anxiety group and in non-anxiety group of cancer patients.

Drawing characteristics		Anxiety self-evaluation results		*χ* ^2^ value	*P *value
		Anxiety	No anxiety		
A1	Yes	13 (48.1)	100 (71.4)	5.607	0.018
	No	14 (51.9)	40 (28.6)		
A3	Yes	15 (55.6)	98 (70.0)	2.158	0.142
	No	12 (44.4)	42 (30.0)		
A4	Yes	13 (48.1)	47 (33.6)	2.089	0.108
	No	14 (51.9)	93 (66.4)		
A6	Yes	10 (37.0)	28 (20.0)	3.738	0.053
	No	17 (63.0)	112 (80.0)		
A7	Yes	13 (48.1)	45 (21.1)	2.558	0.110
	No	14 (51.9)	95 (67.9)		
A9	Yes	13 (48.1)	96 (68.6)	4.165	0.041
	No	14 (51.9)	44 (31.4)		
A12	Yes	13 (48.1)	37 (26.4)	5.090	0.024
	No	14 (51.9)	103 (73.6)		
A13	Yes	5 (18.5)	6 (4.3)	7.318	0.021
	No	22 (81.5)	134 (95.7)		
A16	Yes	11 (40.7)	30 (21.4)	4.557	0.033
	No	16 (59.3)	110 (78.6)		
A17	Yes	12 (44.4)	39 (27.9)	2.936	0.087
	No	15 (55.6)	101 (72.1)		
A19	Yes	10 (37.0)	14 (10.0)	11.338	0.001
	No	17 (63.0)	126 (90.0)		
A25	Yes	13 (48.1)	90 (64.3)	2.494	0.114

**Table 4:** Logistic regression analysis examining role of S-HTP drawing characteristics in predicting anxiety.

Included painting characteristics	*b*	*S*_*b*_	Wald *χ*^2^	*P*	OR	95%OR	
						Lower	Upper
A1	-2.641	0.740	12.732	≤0.001	0.071	0.017	0.304
A3	-1.046	0.634	2.719	0.099	0.351	0.101	1.218
A4	1.607	0.656	5.995	0.014	4.987	1.378	18.048
A6	1.619	0.684	5.599	0.018	5.050	1.321	19.315
A9	-2.327	0.697	11.159	0.001	0.098	0.025	0.382
A13	3.050	0.991	9.482	0.002	21.120	3.030	147.185
A17	0.901	0.604	2.226	0.136	2.463	0.754	8.049
A19	2.295	0.742	9.566	0.002	9.922	2.318	42.478
A25	-3.227	0.909	12.599	≤0.001	0.040	0.007	0.236
Constant	1.202	0.821	2.143	0.143	3.326		

**Table 5:** Comparison of the incidence of anxiety in cancer patients by SAS and S-HTP.

SAS	HTP test		Total
	Anxiety	No anxiety	
Anxiety	15	12	27
No anxiety	7	133	140
Total	22	145	167

**Table 6:** Univariate analysis of SAS scores in different subgroup.

Items	Cases	Score ($\overset{-}{\chi }\pm s$)	Test-value	*P* value
Type of Cancer				
leukemia	2	45.00±0.00	F=1.590	0.107
bladder cancer	1	31.25		
nasopharyngeal carcinoma	27	43.24±7.06		
lung cancer	85	42.41±7.78		
cervical cancer	15	46.83±6.63		
colorectal cancer	13	38.75±7.09		
lymphoma	6	38.13±8.79		
ovarian cancer	2	47.50±0.00		
breast cancer	3	49.17±10.10		
esophageal cancer	6	40.00±8.51		
gastric cancer	4	43.75±10.10		
malignant thymoma	3	38.75±4.33		
Metastasis				
yes	54	43.33±7.86	t=0.929	0.354
no	113	42.15±7.66		
Pain degree				
none	88	42.02±6.66	H=5.508	0.138
mild	55	41.85±9.22		
moderate	20	46.69±7.21		
serious	4	47.50±5.77		
Comorbidity disease				
yes	42	40.54±9.26	t'=-1.707	0.093
no	125	43.20±7.05		
Tumor stage				
I	5	45.25±8.22	F=0.613	0.654
II	10	45.13±11.87		
III	45	42.61±7.88		
IV	53	42.62±7.72		
undetermined	54	41.64±7.68		

## Data Availability

The datasets used and/or analyzed in the present study can be available from the corresponding author upon reasonable request.
